# Analysis of postpartum and other depression detection using machine learning and deep learning techniques: a systematic review

**DOI:** 10.3389/frai.2026.1849458

**Published:** 2026-08-18

**Authors:** Revathy P., Akila Victor

**Affiliations:** School of Computer Science and Engineering, Vellore Institute of Technology, Vellore, India

**Keywords:** deep learning, machine learning, multi-source data, postpartum depression detection, transformer models

## Abstract

Postpartum depression is a major pregnancy-related mental health issue. Currently, computational intelligence research is increasing rapidly for early detection. This systematic review provides a detailed analysis of previous studies based on datasets, models used in machine learning and deep learning, feature selection methods, preprocessing techniques, and performance metrics. Scholarly works were identified across various sources, and they focused on NLP approaches, multi-source data, and forecasting models. Findings show that the research area remains underdeveloped, with most investigations relying on small or single-site data and a small set of features. Although some recent studies have introduced CNN, LSTM, and Transformer models. There is still research on the use of rarely used multimodal, multilingual, and real-time data. Moreover, there are insufficient XAI methods that impede healthcare trustworthiness and real-world implementation. The study highlights critical gaps in medical testing, model generalization, standard comparisons, and diversity in datasets. Future research aims to develop an understandable, scalable PPD detection model. Upcoming investigations should prioritize large-scale, multi-source datasets, incorporate XAI and improved feature representation, and healthcare-oriented assessments.

## 1 Introduction

Depression is a common mental illness that disrupts one’s normal functioning in day-to-day activities for a prolonged period. Depression is not a common mood swing; it affects one’s relationships, official work, and interests in things that one has enjoyed before. Almost 280 million individuals have been affected by depression. Women are affected more than men. Over 700,000 individuals pass away because of suicide each year (Depressive Disorder, 2025). There are different types of depression disorders; Bipolar disorder is one of the persistent psychological states defined by fluctuating phases of elevated mood (Li et al., 2022), as well as distinguished from sand 20renia using machine learning techniques (Hwang et al., 2025) Seasonal affective disorder is a chronic variant of depression that generally arises in certain periods, like fall and winter, and goes away when the season ends (Praschak-Rieder and Willeit, 2003). Major Depressive Syndrome remains an intricate and diverse psychological disease affecting millions globally (Sun et al., 2023). According to the World Health Organization, globally, one in ten pregnant women and thirteen out of a hundred new mothers suffer from mental illness. In developing countries, the rate is higher—nearly 15% during pregnancy and 20% after giving birth. It may lead the mother to commit suicide when it gets worse, and also it affects the child’s development (Perinatal Mental Health, 2025). From the above, depressive disorder, major depressive illness, and perinatal depression occur within 12 months after delivery and need more concern globally (Moreira et al., 2019; Zhang et al., 2021; Oğur et al., 2023).

The Treatment and medication for depression include antidepressant drugs like (SSRIs)- Selective serotonin reuptake inhibitors, which are most commonly used during postpartum depression that occurs during pregnancy, and some of the psychotherapies have illustrated efficacy, such as CBT-Cognitive Behavioral Therapy, Group Therapy, Family Therapy, and Interpersonal Psychotherapy, Meltzer-Brody (2011) and Cantwell (2021) which gives the best choice for speedy recovery when taken along with the medications, and also the author discusses the clinical approaches to treating depression during pregnancy.

Some of the research focused on mental health during the COVID-19 pandemic. The changes in lifestyle due to the Quarantine period, the reliance on social media platforms, and the loss of loved ones contributed to significant mental pressure for many individuals. Handling these situations became challenging for everyone (Dunjic-Kostic et al., 2025). To detect emotional distress, machine learning algorithms and a convolutional neural network are used in Deep Learning (Benito et al., 2024; Kodati and Dasari, 2024; Zogan et al., 2024).

Traditional machine learning techniques, such as Random Forest, Extreme Gradient Boosting (XGBoost), Support Vector Machine (SVM), Naïve Bayes, Logistic Regression, k-Nearest Neighbors (kNN), and Multi-Layer Perceptron (MLP) (Zhou et al., 2024; Benito et al., 2024; Zhu et al., 2023) have been used to detect and improve depression accuracy.

Deep learning techniques such as CNN, Bi-LSTM, VGG-16, Time series with Inception module (Lilhore et al., 2024; Rafiei et al., 2022).

Some of the Explainable AI used are SHAP (SHapely Additive exPlanations), UMAP (Uniform Manifold Approximation and Projection), and Breakdown (Benito et al., 2024; Zhu et al., 2023).

This systematic review provides an extensive analysis of Machine learning and deep learning techniques for postpartum and depression-related prediction, highlighting current methodological gaps along with future research directions.

**Figure 1:**
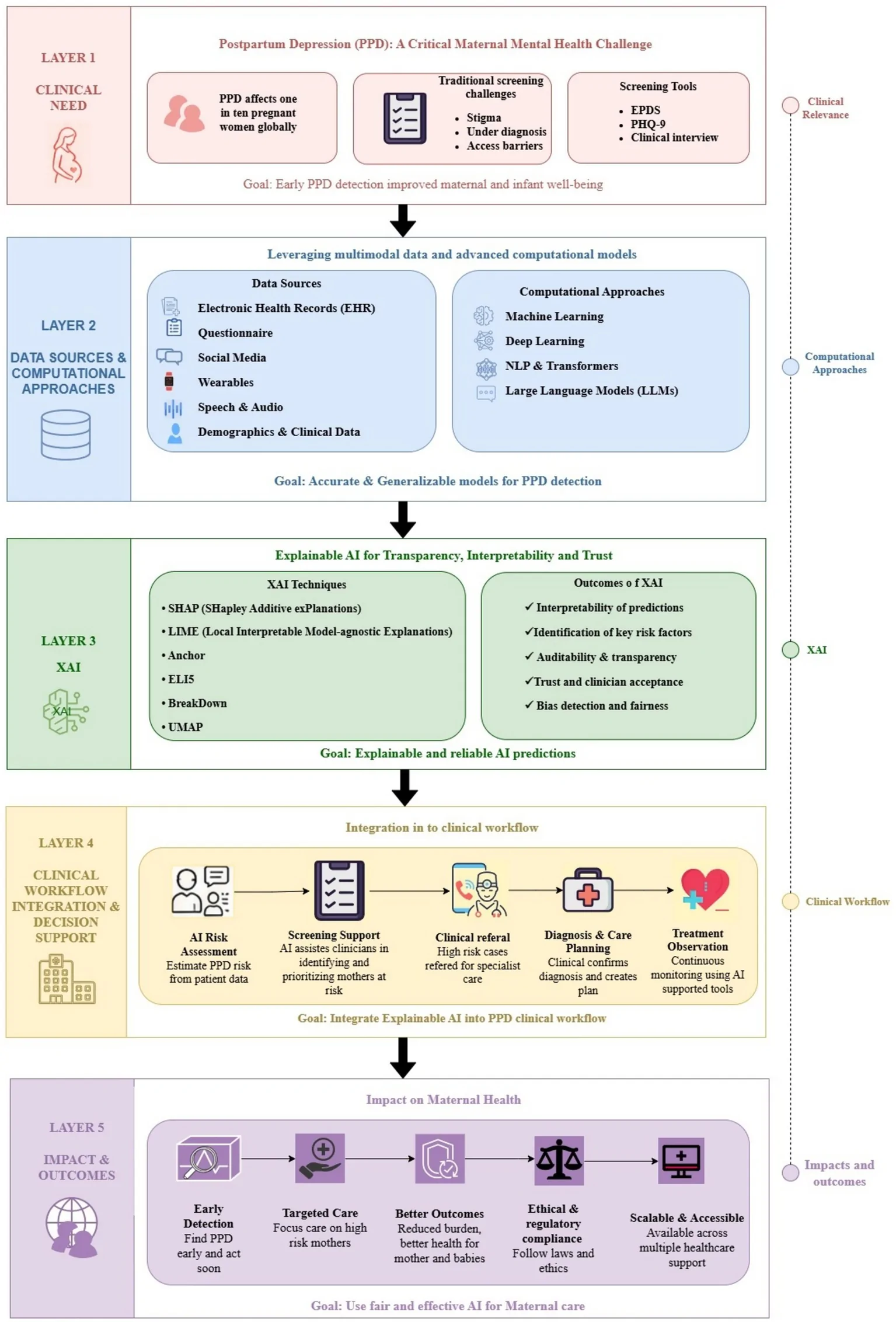
The conceptual framework encompasses clinical need, data sources, computational models, XAI, clinical workflow, and outcomes in AI-based maternity-related emotional well-being.

Despite the rising level of computational studies on postpartum depression screening, a substantial gap remains in methodological improvement and clinical application. To demonstrate how a computational postpartum depression detection system can be adopted within clinical environments, a conceptual model was designed to illustrate five interrelated layers, as shown in Figure 1. The subsequent subsection summarizes previous studies across all five levels to identify where the present research is insufficient for the objective of real-world implementation.

## 2 Methodology

**Figure 2:**
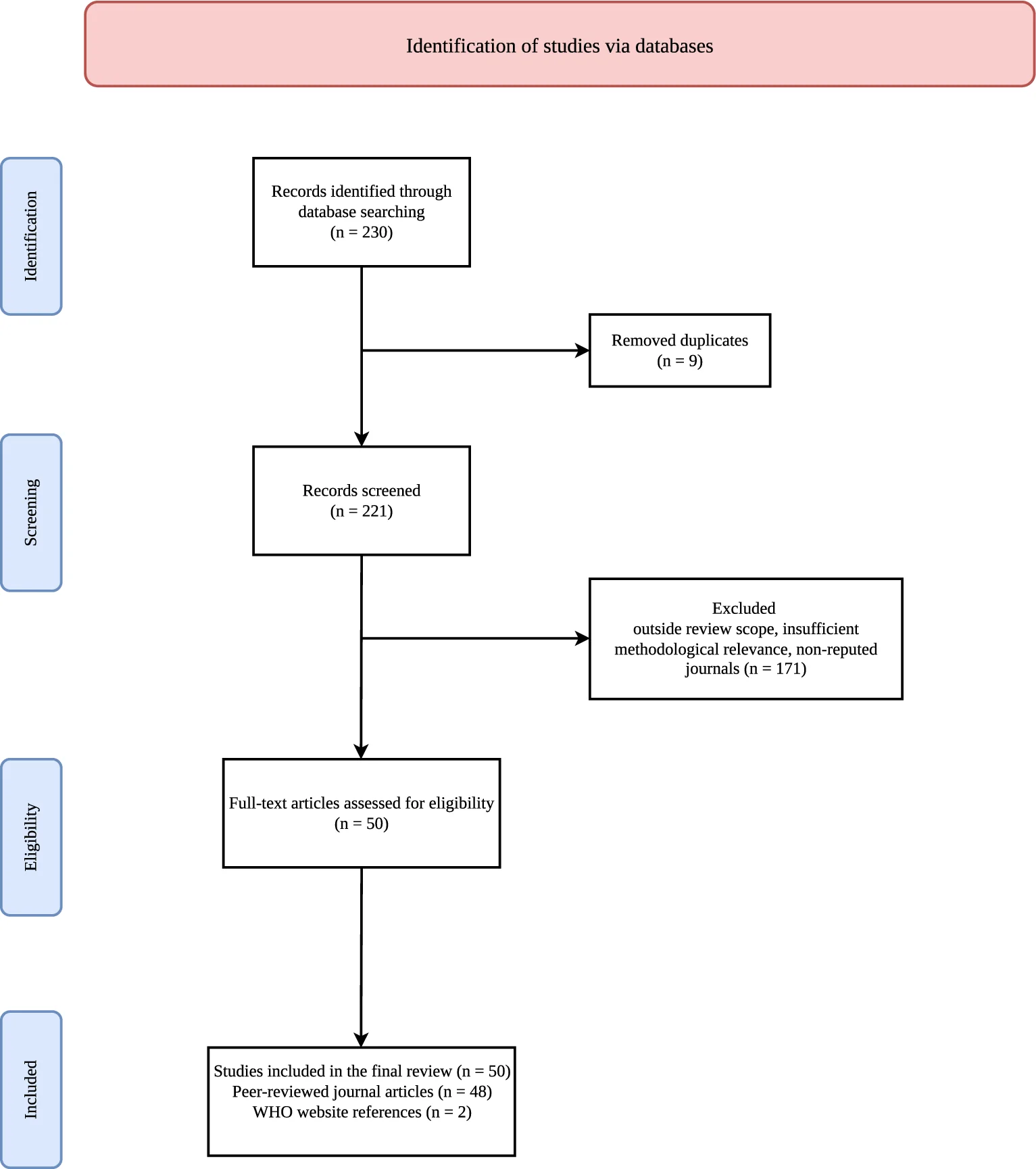
PRISMA flow diagram.

The present literature review followed the PRISMA 2020 guidelines (Preferred Reporting Items for Systematic Reviews and Meta-Analyses) and used a systematic and transparent study screening process to identify and retain relevant studies on computational techniques for PPD detection. The descriptions that are provided in sections 2.1–2.3 and illustrated in Figure 2.

### 2.1 Search strategy

**Figure 3:**
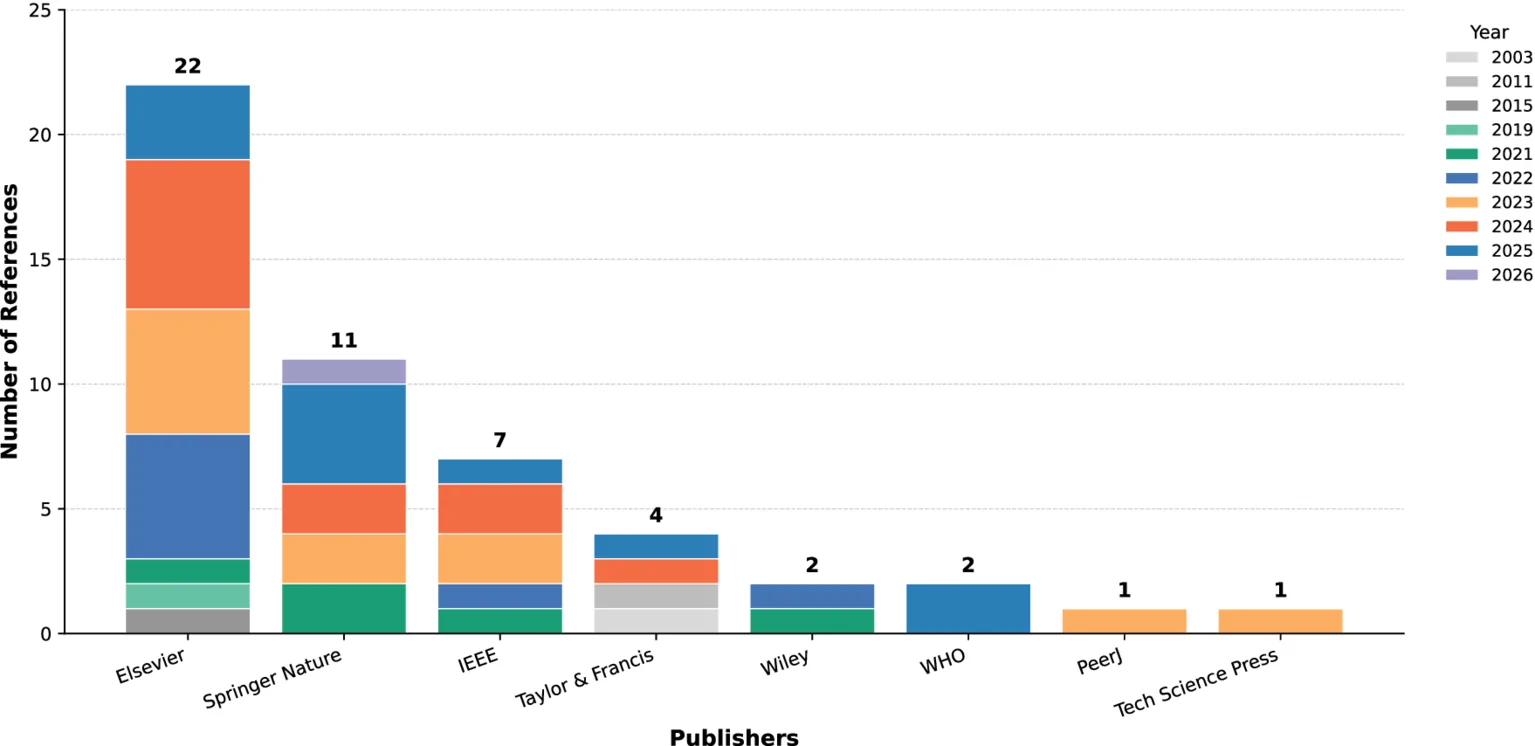
Journal distribution based on count.

A systematic literature search was carried out across 8 web-based repositories and journal platforms: Elsevier, Springer Nature (including BMC and Scientific Reports), IEEE, Taylor & Francis, Wiley, WHO websites, PeerJ, and Tech Science Press. The last search had been performed on January 13, 2026. The following keywords were searched independently and in combination with “mental health,” “depression,” “postpartum depression,” “postnatal,” “perinatal depression,” “maternal mental health,” “pregnancy-related,” and with the technical terms “machine learning,” “deep learning,” and “XAI.” The search was focused on publications published from 2015 to 2026. Praschak-Rieder and Willeit (2003) and Meltzer-Brody (2011) were two earlier studies that were excluded from the structured computational scholarly search and solely included as contextual references in the introductory section. The search strategy focused only on English-language peer-reviewed journal articles and trusted healthcare organizations and websites. Figure 3 demonstrates the distribution of selected studies across journal platforms.

### 2.2 Inclusion and exclusion criteria

Inclusion and exclusion criteria are shown in Table 1.

**Table 1:** Shows the inclusion and exclusion criteria.

Criteria	Inclusion	Exclusion
Subject focus	Research concentrated on depression detection or prediction	Research addressing non-depressive disorders, non-specific psychiatric illnesses, and irrelevant to depression detection
Journal source	Trusted healthcare organization’s webpages or peer-reviewed scholarly publications	Book chapters, conference abstracts, preprints, and research published in non-reputable or predatory journals
Language	Only the English language	Studies not in English
Methodology	Studies employing machine learning, deep learning, or natural language processing strategies	Studies utilizing only medical, statistical, or subjects beyond the computational focus for this study

### 2.3 Study selection

The primary database search strategy retrieved 230 publications, from which 9 duplicates were excluded, leaving 221 distinct records for eligibility screening. The following 171 records were excluded on three bases: conditions other than depression, unrelated psychiatric illnesses, published in non-reputed journals, and limited methodological relevance to the review scope. The 50 retained studies, including 48 peer-reviewed scholarly articles and 2 WHO webpage references, were included in the final study selection. A PRISMA workflow diagram demonstrating the complete study selection approach is shown in Figure 2.

### 2.4 Risk of bias assessment

The methodological quality of all empirical predictive model studies was evaluated using PROBAST+AI (Moons et al., 2025), the latest revision of PROBAST, specifically designed to assess AI- and ML-based predictive models in clinical applications. Using 34 evaluation questions, the assessment covered four domains: D1-Participants and data sources, D2-Predictors, D3-Outcome, and D4-Analysis. Each domain was rated accordingly, with risk of bias rated as low, unclear, high risk, and applicability rated as low concern, unclear concern, great concern. The overall risk was calculated as the highest risk across all four domains. The complete evaluation is provided in Supplementary Table S1.

## 3 Related works

Malhotra and Jindal (2022) Performed a study on the systematic analysis of deep learning strategies for detecting depression, thoughts of self-destruction, and self-injury using online user input from web-based communities. Previous studies have mainly focused on standard machine learning algorithms. This study uniquely concentrates on non-clinical data from digital media platform text, with the main aim of highlighting the deep learning techniques used to identify mental distress, i.e., depression. Techniques like Convolutional Neural Networks, recurrent neural networks, Bidirectional Long Short-Term Memory, Gated Recurrent Unit, along with the transformer models like Bidirectional Encoder Representations from Transformers, are discussed in the context of the multiple data sources used for online text like Twitter, Reddit, Weibo, CLPsych, and mention the challenges from the overall perspectives.

Gadzama et al. (2024) have focused on the research overview of Machine Learning techniques and deep learning techniques for understanding and detecting depression, providing insights into efficient techniques used in deep learning and machine learning techniques to boost the prediction, detection, classification, and diagnosis of depression, and also discussed the Existing publications sources, the data collection and Data sources, the possibilities of the performance outcome, limitations, and overview of each techniques how well it will work on the existing work. This study summarizes the most widely used Deep Learning techniques: Convolutional Neural Network, Long Short-Term Memory, Bidirectional Long Short-Term Memory, and Recurrent Neural Network. Mainly, the survey says the most widely used social media data are Twitter, Facebook, and Reddit.

Alkhateeb et al. (2026) the most recent study in this area presented the PRISMA-ScR guidelines. The findings from this comprehensive study, which described how diverse methods were implemented in empirical studies of PPD that utilized AI, covered a wide range of ML algorithms and discussed methodological considerations associated with preprocessing techniques and validation techniques for clinical datasets. However, the review did not expand to DL structural analysis, a structured XAI framework, a comprehensive review of non-clinical modalities, or a medical implementation framework integrating computational outcomes into real-world application. Moreover, the limitations in the previous study, such as Malhotra and Jindal (2022) on online social media-based depressive disorder detection, lack clinical validation, and Gadzama et al. (2024) provided a generic ML and DL review excluding rigorous research methodology. Overall, this results in several significant analytical components that are unexplored, which the present study addresses. The present study enables an improved insight into the broader context of depressive disorder, major depressive disorder, mood disorder, maternal mental health, and COVID-related depressive psychological distress. This analysis allows cross-diagnostic methodological comparison that highlights which AI-based methods generalize across depression-related disorders. Furthermore, a systematic review focused on DL architectures, such as integrated CNN with BiLSTM models, attention mechanisms, EDA-based ensemble models, and emerging methodologies (including transformer-based techniques and natural language processing), provides methodological insights into architectures inadequately explored in existing studies. Additionally, the present study provides a risk-of-bias and applicability assessment based on PROBAST+AI, offering a methodologically focused insight that was lacking in the existing reviews, which did not include a standardized methodological quality assessment. Furthermore, a systematic classification of various XAI techniques SHAP, LIME, ELI5, Anchor, UMAP was contributed, together with a systematic analysis of clinical interpretability vs. model performance trade-offs, Ethical concerns, bias, fairness, and generalizability, challenges associated with these specific methods, as described in sections 4.2–4.2.3. In addition to the clinical dataset, which is prevalent in earlier studies, evolving non-clinical data types, namely online text data, EEG signals, MRI, federated learning models, and IoT-based wearable devices, are systematically addressed as a distinct methodological group. Moreover, a five-layer computational framework is shown in Figure 1, which connects clinical relevance, data sources, and computational approaches, XAI, Clinical workflow, and outcomes, providing a translational insight lacking in previous studies.

### 3.1 Machine learning methods used for postpartum depression detection

The findings reported by Zhang et al. (2021) developed and validated the uncertainty of postpartum depression. The main motive of this investigation is to find an efficient way of predicting and diagnosing the mental health of a woman after giving birth to a child. The data are collected from two regions: one from WCM during 2015 to 2018, from a single facility, approximately 15,000 women, and the other from NYC-CDRN during 2004 to 2018, around 54,000, extracted from various sites. Initially, the study starts with data preparation. In this approach, the author used the sequential forward selection method for feature selection and greedy search for hyperparameter optimization. The technical method used here is a Supervised Machine learning algorithm, such as Random Forest, Regularized Logistic Regression, Extreme Gradient Boosting, Decision Tree, and Multilayer Perceptron. Among these, the Regularized Logistic Regression performed well on the Area under the Receiver Operating Characteristic Curve, yielding 0.937 and 0.886 for the WCM and NYC-CDRN datasets.

Expanding the EHR-based technique (Zhang et al., 2021) to a significantly greater cohort, researchers (Amit et al., 2021) developed a machine learning algorithm to evaluate the vulnerability of postnatal depressive disorder using the UK Electronic Health Record and used the female participants of 2,66,544, ranging from the age Group of 18–45, who were collected during the years 2000 to 2017. From this, the features were selected based on socio-economic measures, demographics, pregnancy complications, medical history, and use of medical services. XGBOOST was applied with evaluation conducted using regional, time-based, and randomized partitions. The results obtained from this algorithm for area under the curve (AUC) values in the range of 0.72 to 0.74, in the case of early prediction based on prior features, produced diminished effectiveness, significantly when merging with the Standardized questionnaire (EPDS), the probability measures were raised from 0.805 to 0.844, and enhanced sensitivity at a specificity of 0.80. The model explanation was highlighted using SHAP.

Andersson et al. (2021) gathered data from Sweden (*n* = 4,313) to assess various machine learning algorithms for predicting postpartum depression. The evaluated algorithms are Distributed Random Forests, LASSO Regression, Gradient Boosting, Ridge Regression, Extremely Randomized Trees, Naïve Bayes, and Stacked Ensembles. The performance was evaluated based on accuracy, sensitivity, specificity, positive predictive value, negative predictive value, and AUC. The result of Extremely Randomized Trees performed well, yielding 73% accuracy, 72% sensitivity, 75% specificity, and an AUC of 0.81.

In the methodological approach, Oğur et al. (2023) proposed a hybrid approach for feature selection and classification to detect anxiety and depression during the perinatal period. In this study, all the data were collected based on the questionnaire data from the original data of pregnant women in the Sakarya University Research Hospital. The accepted participants were asked to complete the forms based on the SDVF (Sociodemographic and Clinical Data Form), EPDS (Edinburgh Postnatal Depression Scale), and PASS-TR (Perinatal Anxiety Screening Scale), which were created by specialists for diagnostic purposes. They have used 147 features after all the preprocessing. The combined approach of the Marine Predator Algorithm and K-Nearest Neighbors yields optimal results for feature selection and classification; as a result, the accuracy achieved was 0.9811, providing insight before feature selection is applied. It does not provide accurate results as a hybrid approach, and the chi-square test was also conducted, yielding a *p*-value of 0.9770. Advancing feature selection-based strategies (Qi et al., 2025) conducted a follow-up study to predict and verify the PPD in perinatal women using different selection techniques. The data were collected from the maternity hospital, including the state of mental illnesses, and the EPDS assessed the initial outcome. The author used seven Feature selection methods for handling complex data, and for determining the most significant predictors in predictive analytics, they are 3 changes in stepwise regression (forward, backward, bidirectional) for finding important variables, LASSO Regression determines a subset belonging to the appropriate feature, and prevents overfitting, Two variants of Random forest utilized are Mean Decrease Accuracy, Mean Decrease Gini, were selected for robustness in managing nonlinear correlation interaction among predictors, the sequential elimination method of Support Vector Machine- Recursive Feature Selection is used for enhance feature subsets based on architecture performance ensuring relevance for the classifier were utilized to identify 11 key features. Those final predictors were used in the training and validation sets. Greedy search with 5-fold cross-validation was applied to measure 6 machine learning techniques: Decision Tree, Random Forest, Logistic Regression, Support Vector Machine, XGBOOST, and Artificial Neural Network. The performance metrics included sensitivity, specificity, AUC, F1 score, calibration plot, Brier score, and decision curve analysis. The results provide the best logistic regression for AUC (0.801, 0.858) and Artificial Neural Network AUC (0.787, 0.844). Most importantly, uses the SHAP to enhance interpretability. The author developed a tool to facilitate initial diagnosis and decision-making processes.

Researchers (Nasim et al., 2024) introduced a machine learning framework for diagnosing maternal mood disorders. The data were gathered from a hospital via a Google Form, with 1,503 entries, and underwent a preprocessing technique. The data is split into training and testing sets using an 80:20 ratio and k-fold cross-validation. The machine learning models applied are also used for comparison. The author introduced an MDKR meta model in which data are initially handled using Decision Trees, KNN, and Random Forest; the resulting outcomes are fed into a Multi-Layer Perceptron to make the final decision. This approach enhances the overall performance. Responding to the requirement for model clarity, Shivaprasad et al. (2024) introduced an Explainable AI architecture to estimate maternal depression risk and correlated it with self-harm incidents. The dataset used in this study is a freely accessible Kaggle dataset containing 1,503 mothers after childbirth with ten features, including emotional and behavioral features. The study highlighted clarity and explainability through the combination of four explainable AI methods, LIME, SHAP, ELI5, and Achor, to determine the most significant risk factors. Data preparation and one-hot encoding were used to organize data for the analysis, and various predictive models were evaluated, including logistic regression, tree-based models, random forests, KNN, adaptive boosting, Light Gradient Boosting Machine, Extreme Gradient Boosting, Categorical Boosting, and a tailored stacking ensemble model. Among these, KNN and the Tailored Stacking Ensemble Model attained the best accuracy of 97%, indicating strong precision and sensitivity. XAI evaluations pinpoint the major determinants. The authors concluded that incorporating ML algorithms and XAI techniques strengthens trust and medical understanding and preliminary detection of postpartum depression, facilitating individualized psychological treatments.

Diverging from the studies based on questionnaires, researchers (Moreira et al., 2019) developed a strengthened algorithm for monitoring mental health signals. Data is collected from Maternity School Assis Chateaubriand of the Federal University of Ceará, Fortaleza, Brazil using a wearable IoT device and stored in the private cloud based on clinical and sociodemographic data for early prediction of postpartum depression along with other risk factors related to pregnancy by assessing with machine learning algorithms such as Decision Tree, Support Vector Machine, K-Nearest Neighbors, and Ensemble methods (bagging and boosting), whereas bagging trains multiple Decision Trees and predicts based on the majority of voting, boosting Sequentially corrects error based on past one, for validation 10 cross fold validation is used, the performance and assessment result based on the accuracy, True Positive Rate, False Positive Rate and also calculated through Area under the Receiver Operating Characteristic curve(AUC-ROC). This study presents comparative results on the accuracy of all four algorithms across different scenarios, with symptom bagging achieving approximately 95% accuracy.

Table 2 illustrates the limitations and other key findings from the Machine Learning-based PPD research discussed in section 3.1.

**Table 2:** Classified by data type, key findings, and limitations from postpartum depression using ML.

Study	Methodology	Data type	Key findings	Limitations
Studies based on EHR data				
Zhang et al. (2021)	Supervised ML algorithm	EHR data	Regularized logistic regression is the most effective performing model	Challenges are low positive predictive values, a lack of widely used tools like EPDS, and PHQ
Amit et al. (2021)	XGBOOST	EHR data	EHR with EPDS improved the result	Missing data, Single algorithm dependence
Studies based on questionnaire data				
Andersson et al. (2021)	Machine Learning algorithms	Clinical and Questionnaire data	The Extremely Randomized Tree performed well	Data Imbalance and Generalizability
Oğur et al. (2023)	MPA + KNN	Questionnaire data	The hybrid approach gives the best optimized result	Generalizability, Comparative studies can be made using advanced classifiers
Qi et al. (2025)	Supervised machine learning algorithms	Questionnaire data-based on demographic characteristics, mental health history, psychosocial factors, and physiological measures	Logistic Regression and Artificial Neural Network give the best performance results	Lack of external validation, Risk of overfitting, and limited hyperparameter tuning
Nasim et al. (2024)	MDKR model	Questionnaire data (Google Form)	11 machine learning algorithms were compared; among those, MDKR performed well	Limited Data, Higher runtime, complex model
Shivaprasad et al. (2024)	Machine learning algorithms, XAI techniques	Questionnaire data from Kaggle	Among all the ML algorithms, KNN and stacking ensemble give the best outcome	Insufficient sample size, self-reported records bias, and scarcity of external validation
Studies based on IOT				
Moreira et al. (2019)	Four machine learning algorithms	Clinical and sociodemographic data	Bagging gives an efficient outcome	Focused on only a few other risk factors like diabetes, hypertension, obesity, and ICU admission. Smaller dataset

Based on data type, where further classified into three categories: EHR, Questionnaire, and IoT-based data. Less sophisticated prediction models have been shown to generate equivalent or stronger performance than more advanced prediction methods. For instance, Zhang et al. (2021) observed that regularized logistic regression (AUC: 0.937) was the highest-performing model among the other algorithms in the study, including MLPs. Likewise, Qi et al. (2025) reported that the Logistic Regression (AUC: 0.858) produced better model performance than ANN models (AUC: 0.844). The outcome of these studies indicates that the use of simplified predictive models may mitigate the threat of overfitting when generating reliable outcomes from smaller, clearly defined medical or EHR data collections.

Alternatively, Oğur et al. (2023) obtained the best performance of 0.9811 using a hybrid metaheuristic-classifier model on questionnaire data, suggesting a benefit to the utilization of fusion and integration of classification and feature optimization with more diverse multidimensional questionnaire data than one data modality alone. Despite sequential forward selection, MPA showed stable performance throughout all categories and improved outcomes, therefore suggesting that feature engineering may be more significant than the data modality from which it is derived.

Some methodological limitations persist, making direct comparisons between investigations challenging. Researchers (Qi et al., 2025) used the EPDS only as the target label. Conversely, researchers (Amit et al., 2021) showed that EPDS was incorporated as an input variable. Thus, the AUC values observed by each study cannot be compared on the same basis since they are derived from distinct instances of predictive tasks.

### 3.2 Methodological review of deep learning techniques

Gopalakrishnan et al. (2023) have proposed a deep learning technique to enhance the detection of postpartum depression. The data is collected from SRM Medical College and Research Center in the form of both a physiological assessment form based on PDSS and social media data (Facebook), collected via the API. Data cleaning and processing were initially performed on 949 records, and the cleaned data were 631 records. They were put forward to the ASHN Model, which consists of 4 attributes: sentimental words, depressive symptoms words, psycho-linguistic style, and Ruminative response style. The hyperparameters of the model include 50 epochs, the Adam optimizer, a dropout rate of 0.3, a learning rate of 0.5, a batch size of 64, and evaluation.

Advancing from text-based online social media datasets into a multi-modal technique (Lilhore et al., 2024) introduced an enhanced integrated deep learning model by incorporating a CNN-based transfer model and an improved Bi-LSTM. The author used multimodal text-based and speech data from the UCI dataset, comprising 1,550 textual and 195 audio files, enabling multi-feature pattern learning through a Convolutional Neural Network feature extraction and temporal sequence via an interpretable Bi-LSTM with attention mechanisms. The combination of both data achieved a better predictive accuracy.

Moving to a biomarker-based data modality, researchers (Gopalakrishnan et al., 2025) developed an ensemble-based deep learning model for forecasting the severity of pregnancy-related depression, utilizing electrodermal activity Signals captured by smart wrist devices. The research focused on the clinical need for ongoing contactless tracking of pregnancy stress during the perinatal period. Data were collected from 189 respondents registered at SRM Medical College with ethical clearance, and depression scores were used for annotation. The preprocessing pipeline includes movement artifact detection, utilizing an autoregressive model, signal slicing through a non-overlapping window, and variable frequency derivation using VFCDM and RF-based feature selection. The proposed model integrated multiple deep networks in a stacked ensemble arrangement, utilizing both subject-dependent and independent evaluation through LOOCV. Performance comparison across the standard datasets (WESAD, CLAS, VerBIO) established an improved precision of 93.87%, better than baseline models, artificial neural network, k-nearest neighbor, random forest, and decision tree.

Table 3 presents the limitations and other key findings from the above-discussed study in section 3.2.

**Table 3:** Data type, key findings, and limitations from postpartum depression using DL.

Study	Data type	Algorithm used	Evaluation metrics	Key findings	Limitations
Gopalakrishnan et al. (2023)	PDSS questionnaire and Facebook posts	Attribute Selection Hybrid Network (ASHN), RNN with Post-level attention.	Precision 78.77, Recall 72.94, F1 Score 74.77, Accuracy 75.74	ASHN predicted higher than the baseline	Limited data, only on the English post, and a lack of external validation.
Lilhore et al. (2024)	UCI PPDD dataset (text and audio)	CNN-based transformer model, along with improved Bi+LSTM plus attention	Accuracy 98.1%, Precision 98.7%, F1-Score 98.4%, Recall 98.4%, MSE 0.01071	Achieved significant accuracy, along with identifying multimodal risk factors	Small and uneven dataset, confidentiality and ethical constraints for audio data, and lack of external cross-validation
Gopalakrishnan et al. (2025)	EDA signals data	Ensemble-based deep learning	Precision 91.54%, Recall 92.19%, Accuracy 93.87%, F1- Score 92.19%, Root mean squared error 0.31, Mean absolute error 0.24.	The proposed algorithm performed well when compared with the baseline algorithm	Requires continuous tracking, restricted only to EDA, possible sensor interference, and diverse dermal reactions

The three neural network-based studies, attention-based combined networks, attention-enhanced CNN-BILSTM, and stacked ensemble framework, respectively, obtained significantly distinct performance levels regardless of a similarly complex framework, as Table 3 demonstrates. This distinction is more likely to be associated with the integration of data types compared with model complexity alone. Importantly, despite good model performance, neither of these studies provides an explainability method, suggesting that this is a methodological shortcoming observed across DL architectures in this category, instead of one specific kind of data.

### 3.3 Methods used from NLP techniques

Krishnamurti et al. (2022) presented the detection of risk in pregnancy using NLP methods, where the data is collected from the UPMC healthcare systems, USA, using the MHP mobile application. Additionally, they used the EPDS Scale Questionnaire. The author used several NLP techniques, including Latent Dirichlet Allocation, Linguistic Inquiry, Word Count, SentiWordNet, Word2Vec, and LASSO regression. The time frame was tested, and the performance metrics used here are AUROC based on time, and for open-ended, the AUROC is 0.87. In another study, the author used NLP-driven data collected at a hospital in China, combining it with electrophysiological data, NLP, and traditional Chinese medicine. The data preprocessing and feature extraction used TF-IDF; the algorithm used here is RCO-DRNN, and the performance metrics used in this study are MSE, MAE, R-Square, Accuracy, F1, Precision, Recall, and Specificity (Wang, 2025).

Extending beyond healthcare-based data completely (García-Méndez and de Arriba-Pérez, 2025) The study employs NLP, LLM, and ML by merging for early and precise detection of postpartum depression; the data used here is synthetic data as well as publicly available data from a chatbot and LLM server by using the stream-based ML Classifiers: Logistic Regression, Gaussian Naïve Bayes, Adaptive Random Forest Classifier, Approximate Large Margin Algorithm, Hoeffding Adaptive Tree Classifier. The counterfactual explanation algorithm is used for stream-based explainability. ChatGPT 3.5 focuses on using LLM Techniques for language understanding and communication. These techniques create a live PPD detection based on an open-ended text conversation. The performance metrics used here are Accuracy, Precision, Recall, and F-Measure.

### 3.4 Different techniques for depression detection

The subsequent Tables 4–6 will discuss techniques used to predict depression.

**Table 4:** Analyses focused on depression in earlier periods.

Study	Methodology used	Dataset used	Dataset source	Performance	Limitations
Social media (Text data)					
Ahmad Wani et al. (2023)	CNN + LSTM, Feature representation - Word2Vec, Feature Extraction – TF-IDF	Netvizz, iMacros, and Tweepy strategies To collect depression relevant data	Online social network platforms Facebook, Twitter, and YouTube	Accuracy (0.9901), precision (0.9920), recall (0.9901), f1score (0.9910).	Multimodal can incorporate elements like emoji, audio, and video. Lack of clinical data.
Figuerêdo et al. (2022)	CNN: early fusion, late fusion, Emoticons mapping	Text and emoticons	Reddit e-risk 2017 challenge	Precision-0.76, recall, f1, ERDE	Not generalizable for other platforms, trade-off in detection threshold
Zhang et al. (2023)	CNN with BILSTM.	Text data	Reddit and Twitter dataset	F1 and accuracy: 0.9554 in the Reddit dataset, F1: 0.7984, and accuracy: 0.8031	Limited only to the English language, with high computational complexity because of feature extraction.
Khafaga et al. (2023)	Proposed algorithm: MDHAN, enhanced with Optimization: PSO, GWO, combined as MDH-PWO	Text data	Kaggle Twitter data	Accuracy: 99.86% Precision: 93.45% Recall:91.95% F1: 90.90%	Concentrate exclusively on text data; no long-term evaluation.
Multimodal-based data					
Vandana et al. (2023)	CNN, Hybrid LSTM, Bi-LSTM	Audio/voice recordings, video recordings, and Questionnaire text data.	DAIC-WoZ database from the USC University of Southern California website	Accuracy, Text CNN-0.92, Audio CNN-0.98, Hybrid LSTM-0.80, Hybrid Bi-LSTM-0.88, Precision, Recall, F1-score, Support.	Complex, training duration, Generalization
Patil et al. (2024)	Federated Learning with ExpAPO-DCNN	MRI images, speech signal	MPI-Leipzig-Mind-Brain–Body-dataset	Accuracy: 98.00% Loss: 0.023 RMSE: 0.058 MSE: 0.240 TNR: 97.90% TPR: 96.30%	Binary classification only fails to detect severity.
MRI DATA					
Zhou et al. (2024)	Random forest, feature extraction – GMV, FC, ReHo, (f)ALFF	Clinical neuroimage data (MRI)	The ESCID project, spanning the period from 2019 to 2023, was accepted by the Renmin Hospital of Wuhan University’s ethical committee	AUC-0.802, Sensitivity-0.734, Specificity-0.717	A certain individual took depression medication, which could change cerebral activity, The model needs validation on a larger, standalone dataset. Failed to integrate clinical and psychological attributes for fusion
EEG data					
Seal et al. (2021)	DeprNet	EEG and PHQ-9 Questionnaire data	Self-Collected	Record-Wise (Accuracy: 0.9937, AUC: 0.999) Split-wise (Accuracy: 0.914)	Small dataset, Overfitting may occur.
EMR-based data					
Zhu et al. (2023)	SVM, Logistic Regression, XGBoost, Random Forest, Explainable AI: SHAP, BreakDown	Clinical medical record	EMR Data from a Western China hospital	AUC: 0.838, Positive Predictive Value: 0.810, Negative Predictive Value: 0.834	Insufficient feature selection strategies, class imbalance

**Table 5:** Depression, along with COVID and MDD diseases.

Study	Algorithm used	Dataset type	Dataset source	Performance metrics	Limitations
Based on social media data					
Fkih et al. (2025)	CNN, LSTM, BiLSTM, GRU	Twitter (publicly not available)	Worrycov data	GRU performed well, with Accuracy, F1-score, Precision, and Recall	Dependence on social media data, which is not publicly available.
Kodati and Dasari (2024)	Context-ABT-BiLSTM-CNN	Text data with emotions	Twitter and Reddit data	Accuracy, F1-score, precision, recall	High computational cost, limited only to textual data, and a lack of explainability.
Questionnaire data					
Benito et al. (2024)	XGBOOST, Random Forest, Naïve Bayes, SVM, multi-layer perceptron, novel combination SHAP+UMAP	Questionnaire	COVICAT, data collected from the Hospital, Stress, Anxiety, and Depression	AUROC, Precision, Recall, F1 score.	Can improve the performance, only on California data.
Wearable-based data					
Sarwar et al. (2024)	GRU-based with an attention mechanism, one class model agnostic meta learning (few-shot learning)	Wearable data	Fitbit data	Precision, recall, AUC-ROC, F2 score, accuracy, Negative Predictive Value (NPV)	Limited amount of data for positive cases, device-specific limitations, fixed 2-day window, which may miss critical variations and introduce bias.
EEG-based data					
Sun et al. (2023)	Hierarchical clustering, similar fusion network	EEG Data	Hospital of Tianshui, Gansu, China. Based on DSM-5, PHQ-9	Clustering numbers based on alpha, beta, theta, and delta	Limited analysis of frequency bands and connectivity types, use deep learning for model improvement, and large samples can be used

**Table 6:** Depression detection utilizing optimization techniques and NLP in past years.

Study	Algorithm used	Dataset type	Dataset source	Performance metrics	Limitations
Audio-based studies					
Patil et al. (2023)	Exponential African vulture optimization algorithm, deep maxout network	MRI and Speech signal	https://www.neuroconnlab.org/data.	Accuracy, Sensitivity, Specificity	complexity of fusing the data, data limitations, and lack of severity level
Kaur et al. (2022)	Quantum whale optimization algorithm, classification: KNN, SVM, LDA, Logistic regression.	Speech	DAICWOZ dataset	F1 Score for the depressed class, F1 Score for the non-depressed class, and Error Rate	small dataset, only on speech
EEG-based studies					
Saif Alghawli and Taloba (2022)	Improved Ant Colony Optimization with SVM	EEG data	Collected from the hospital, it is not publicly available	Sensitivity, Accuracy, AUC	Limited data, clinical integration
Text-based studies					
Zhao and Tlachac (2025)	XGBoost with Bayesian optimization	Text data	Used the data from the following paper, StudentSADD: Rapid mobile depression and suicidal ideation screening of college students during the coronavirus pandemic pandemic. Automated construction of lexicons to improve depression screening with text messages	Balanced Accuracy, Sensitivity, Specificity, Precision, F1	Accuracy needs to improve, small data, and a lack of generalizability
Li et al. (2023)	CAFed- Convolutional Neural Network Asynchronous Federated Optimization	Text	Weibo (publicly not available), microblogging platform data, China	Accuracy, Precision, F1, Recall	Small data, data Privacy, need to improve on accuracy.
Akyol (2023)	New improved grey wolf optimization	Text	Reddit	Accuracy, F-Measure, Precision, MCC, Sensitivity	Restricted only to Reddit data, not the use of any XAI
NLP					
Karmen et al. (2015)	DepreSD score, rule-based NLP with a lexicon-based score	Text	Psycho-Babble online forum	Precision, Recall, F-measure, TP, FP, TN, FN	Rule-Based, solely on the shortcoming sentence, not on the reliance phrase.

Despite using a different platform (Ahmad Wani et al., 2023; Khafaga et al., 2023) present strong performance accuracies of 00.01 and 99.86%. Similarly, based on the social media group in Table 3, the results imply that they rely more on common text features than on the specific platform chosen. Even though data types are combined, data integration repeatedly improves the performance based on the findings from Vandana et al. (2023) and Patil et al. (2024) two heterogeneous data investigations achieved the highest accuracy, near 98%, when merging data sources. Since Figuerêdo et al. (2022) and Zhang et al. (2023) report F1-score or precision in place of accuracy; their reported values are measured on a distinct evaluation scale, making direct evaluation among the other analyses less reliable (Table 4).

When analyzing the studies examined in the previous sections across the four approaches, it is important to note that domain-wide developments go beyond the section-level insights reported earlier. A consistent outcome across Machine Learning, Deep Learning, Natural Language Processing, and multimodal frameworks is that model complexity does not consistently predict improved model performance, but rather systematic framework selection; this alignment appears empirically confirmed by the availability of the dataset. Another cross-sectional finding is that XAI approaches are used across all four sections within each study; however, Explainable AI was used as a *post hoc* approach and not as an architecture strategy. This represents a common trend for all four approaches irrespective of dataset category or clinical domain. Further, it is discussed as a research Gap in Section 5. The other significant observation from this study is that it identified the contradictory relationship between reported performance and clinical applicability throughout all four sections. In section 3.4, online social media publications reported the highest accuracy among all the studies present in this review. However, they lacked a validated clinical benchmark completely, whereas in section 3.1, both questionnaire and EHR-based studies showed moderate yet valid classification performance metrics and demonstrated actual clinical applicability by means of clinical result assessments used in large multi-hospital participant groups. This contradictory pattern is characterized by high performance without clinical validation and fair performance with strong clinical reliability, suggesting, at present, the area of study optimizes for measurable predictive accuracy at the cost of medical utility. Future studies should consider external validation and clinical benchmark quality as equally weighted evaluation criteria in addition to model predictive performance while assessing AI-based depressive disorder screening models.

**Figure 4:**
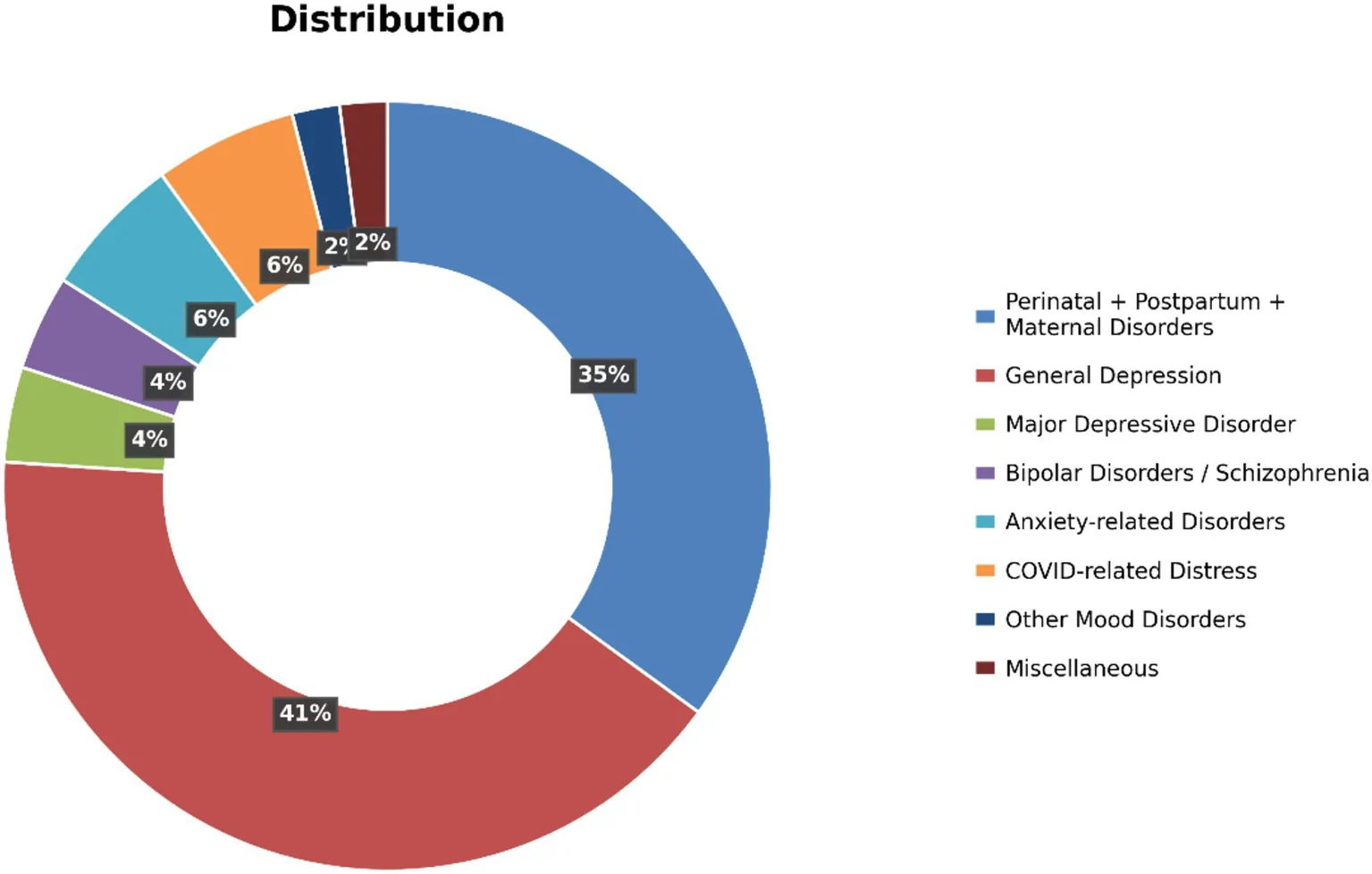
Mental disorder distribution.

Figure 4 presents a visualization of the distribution of each type of mental disorder, as selected for review in this study from the references included here.

## 4 Preprocessing techniques used in text

Detection of depression, the data will have a missing value, noise removal, eliminating unnecessary spaces, removing redundant text, the common NLP-based cleaning using Standard deviation, stemming and Lemmatization, Annotation, Tokenization, stop word Removal (Ahmad Wani et al., 2023; Figuerêdo et al., 2022; Khafaga et al., 2023; Fkih et al., 2025) removing HTML, numbers, labelling, and lowercasing (Zhang et al., 2023; Karmen et al., 2015) The most commonly used data cleaning process in machine learning is treating missing data, using the KNN imputer, which identifies the nearest similar data from the dataset (Benito et al., 2024).

For audio, they have used noise removal and audio segmentation. For the feature, they have employed the Mel spectrogram and MFCCs (Vandana et al., 2023).

### 4.1 Feature selection and feature extraction

Feature selection is one of the most important techniques for selecting the most relevant features to improve efficiency and decrease complexity. It is essential in machine learning.

In Machine Learning, feature selection is mainly focused on Minimum Redundancy Maximum Relevance (Benito et al., 2024). However, in deep learning, this is not necessary because it automatically selects the relevant features and extracts them. As per the knowledge gained from the references, the following feature selection methods are utilized in deep learning: Word2Vec (CBOW with Skip-gram), TF-IDF (Ahmad Wani et al., 2023; Zhang et al., 2023), which almost captures the most relevant vocabulary based on depression. Conversely, Word2Vec embeddings performed well on text. They were then fed into deep learning methods, such as convolutional neural networks and temporal selection like LSTM and BILSTM, without using manual or traditional methods. The feature is extracted (Vandana et al., 2023). Word embedding is used for feature extraction. The following four pretrained embedding techniques are FastText WN, FastText Crawl, GloVe, WN, GloVe Crawl, and are applied to CNN (Figuerêdo et al., 2022). Similarly, the author used the combination of BERT, Glove, FastText, and LIWC for feature extraction, and used the LIWC for feature selection (Fkih et al., 2025). Adaptive Particle Grey Wolf Optimization is utilized for feature selection in this; the author used MDHAN for feature extraction (Khafaga et al., 2023). Moreover, another study used the newly improved grey wolf optimization for selecting the features (Akyol, 2023), mainly when the mental health deals with other issues. It has to extract each feature with its dependencies on diseases. In this, they have utilized a context-aware self-regressive transformer model with BILSTM & CNN, where they newly used a theme-based context like TBT and a rule-based permutation RPB to extract appropriate context data to classify emotions (Kodati and Dasari, 2024).

The study applied an Empath-based word category frequency measure, POS tags (speech and basic structural features), and measures such as word count and message count to extract meaningful patterns. The chi-square feature selection is used to reduce dimensionality. This facilitates preserving the important variables while boosting the model’s effectiveness (Zhao and Tlachac, 2025).

### 4.2 Explainable AI

Model understandability has evolved into a prominent research area because of the growing implementation of machine learning and deep neural learning approaches in healthcare environments. Even though diagnostic accuracy remains a major criterion, medical experts and regulatory authorities require that the outcomes be clear, reviewable, and trustworthy prior to clinical use. This is specifically crucial when it relates to the detection of postpartum depression because incorrect prediction carries severe consequences for the well-being of both mother and infant. XAI refers to a set of techniques that make complex artificial intelligence models understandable, particularly for decision makers, such as physicians, patients, and researchers.

XAI techniques are widely categorized as global, which describe overall computational model performance throughout the data collection, or local, which explicate specific predictions. They are additionally divided into model-independent methods suitable for a wide range of algorithms and model-dependent methods, developed for specific architectures, for detecting depression. The model-independent methods, which are also known as model-agnostic local methods, are broadly implemented due to the heterogeneity of algorithms and data types across the surveyed analyses.

Various XAI methods have been implemented, particularly to improve the transparency of postpartum depression and depression detection as summarized in Table 7.

**Table 7:** Comparison of XAI methods.

XAI methods	Type	Working mechanism	Implementation in PPD	Shortcomings
SHAP	Both global and local	Allocates attribute contribution score using cooperative game theory to every attribute for specific classifications	Amit et al. (2021); Zhu et al. (2023); Benito et al. (2024); Shivaprasad et al. (2024); Qi et al. (2025) to identify significant PPD risk indicators, obtained from EHR and questionnaire data	High computational cost, assumes features to be uncorrelated
LIME	Local	Produces locally reliable regression-based representations near any instance-level predictions	Shivaprasad et al. (2024) to clarify maternal mental health risk predictions from publicly available Kaggle postnatal data	Inconsistent Explanations, vulnerable to data sampling
Anchor	Local	Generates rule-based insights with strong predictive performance	Shivaprasad et al. (2024) to generate clinically meaningful rules for risk assessments in PPD	Excessively precise in high-dimensional datasets
ELI5	Both global and local	Presents a feature of importance, a graphical view, and diagnoses predictive models by examining the algorithm parameters and results	Shivaprasad et al. (2024) to cross-validate risk factors in PPD	Insufficient DL-based Frameworks
UMAP	Visual	Dimensionality reduction	Seal et al. (2021) illustrated EEG-derived feature groups in DeprNet from depression data	Provides graphical perception only and fails to directly clarify the model output.

SHAP (SHapley Additive exPlanations) is the most extensively used XAI method among the analyzed studies. It provides every feature with an attribution score derived from cooperative game theory, providing both global and local clarifications. This makes it appropriate for healthcare datasets where patient-specific predictions and group-level risk factors are equally significant. In PPD detection (Qi et al., 2025). The transparency of their Logistic Regression and ANN models was improved by implementing SHAP to identify and rank the eleven highly significant clinical predictors from questionnaire-based maternal data. Likewise, Amit et al. (2021) showed which medical and sociodemographic features most significantly predicted PPD risk by applying SHAP to clarify XGBoost classification Results based on Electronic Health Records of more than 266,000 female participants in the UK. To analyze classification results from clinical questionnaire-based and EMR-based depression detection models, as in Zhu et al. (2023) and Benito et al. (2024). Furthermore, the SHAP is used in combination with other XAI tools for depression prediction. Although SHAP provides theoretical consistency and supports both linear and non-linear models, it is computationally costly. It assumes that predictor independence may be compromised in clinical data where features are frequently correlated.

LIME (Local Interpretable Model-agnostic Explanations) describes instance-level predictions by modifying the input and applying a simple, linearly structured model to identify which predictors significantly contributed to the result (Shivaprasad et al., 2024). LIME was used in combination with three additional XAI techniques on Kaggle postnatal data. Demonstrating that integrating the various tools generated enhanced, robust predictive factor findings. Despite this, LIME clarifications can be inconsistent, as slight input modifications or sampling differences may generate unstable attribution scores, restricting their robustness in healthcare environments. Moreover, in the same research, Anchor and ELI5 implemented supporting XAI tools. Anchor creates an if-then rule-based justification that ensures classification outcomes under certain conditions, while ELI5 displays predictor importance scores to cross-validate outcomes from SHAP and LIME. Combined, these techniques provide enhanced, comprehensive, and trustworthy insight into PPD risk indicators compared with a standalone technique.

Similarly, Al Kafi et al. (2026) applied LIME-based attribute selection alongside SHAP in a multi-stage XAI architecture for PPD identification, showing that LIME selected 29 key features and achieved 93.2% accuracy while demonstrating higher threshold consistency than SHAP over a broader range of feature sets.

UMAP (Uniform Manifold Approximation and Projection) provides a diverse transparency function from the above techniques. Instead of describing individual predictions, UMAP reduces the high-dimensional attribute space to two or three aspects for graphical examination, enabling researchers to detect inherent clusters, data patterns, and possible outliers in the model input (Seal et al., 2021). UMAP is integrated with a novel SHAP-UMAP approach within a DeprNet architecture to display EEG-extracted feature groups, separating depressed from non-depressed individuals. UMAP provides useful perception into the framework of trained models; it does not explicitly elucidate model predictions and thus serves as a supportive, rather than a standalone, explainability tool.

#### 4.2.1 Clinical interpretability vs. model performance tradeoffs

A primary trade-off occurs between architecture complexity and explainability in healthcare-related artificial intelligence. For well-defined datasets, such as questionnaires and EHRs, interpretable machine learning algorithms, enriched with explainable AI, have achieved accuracy equivalent to that of advanced neural network-based models. In postpartum depression detection, for instance, SHAP-based logistic regression, Random Forest, and XGBoost models demonstrated strong or improved outcomes (Amit et al., 2021; Qi et al., 2025; Al Kafi et al., 2026). These findings indicate that if data are well-defined and clinically significant, transparency could be maintained without sacrificing classification effectiveness.

Conversely, Deep learning frameworks frequently perform better on unstructured data, such as online platform text, voice data, and EEG signals, yet have limited fundamental interpretability. As a result, *post hoc* XAI approaches like SHAP and LIME are important for enhancing clarity and clinical reliability. However, insights from transparent techniques are a simplification of the model response instead of causal support and must be analyzed carefully, specifically in emotional well-being applications. Generally, the explainability performance trade-off relies mainly on the data type, with structured data collections preferring explainable models and raw data leveraging more sophisticated deep learning techniques.

#### 4.2.2 Ethical concerns in healthcare AI

The implementation of an AI-based maternal depression screening tool creates significant ethical concerns. Due to this technology, healthcare facilities can use XAI techniques such as SHAP and LIME (Shivaprasad et al., 2024), which means it is important that healthcare providers make use of tools to understand, evaluate, and when required, modify AI-generated PPD risk assessments (Al Kafi et al., 2026). Furthermore, XAI is implemented in the healthcare environment and is necessary for creating reliable models and introducing them to healthcare recipients, and as a result, healthcare recipients as well as healthcare providers can challenge AI-generated evaluations of their risk for postnatal depression (Blackman and Veerapen, 2025).

#### 4.2.3 Bias, fairness, and generalizability

Algorithmic bias is a significant concern in explainable AI implementations for PPD detection, yet this area has scarcely been explored. Several databases analyzed in this study were acquired from a one-site, monolingual, or demographic-based similar groups—Swedish (Andersson et al., 2021), UK (Amit et al., 2021), and the US (Zhang et al., 2021) study groups, which were also shown by Dang et al. (2024). The researchers conducted a comprehensive review of the effects of machine-learning bias on the prediction of depressive disorder across four nations. They found that widely used machine learning approaches exhibit bias in their likelihood of generating diverse findings for different cohorts, and no standalone algorithm achieves fairness in results among social groups.

**Figure 5:**
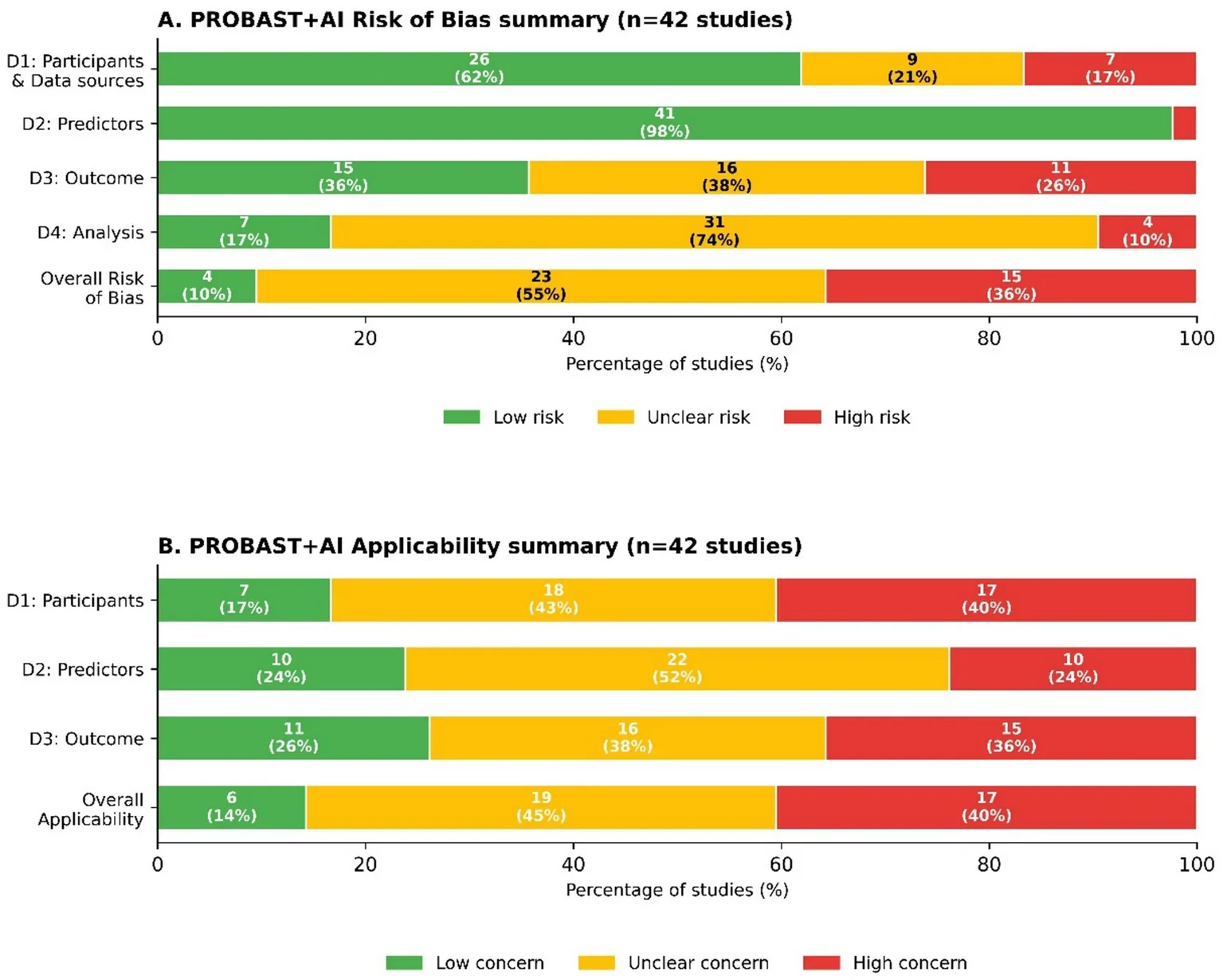
PROBAST+AI risk of bias and applicability summary.

The PROBAST+AI performed across all empirical studies demonstrates structured evidence for the methodological trends identified across the subsections 3.1–4.2.3. D2- Predictors were classified as low-risk assessments in 41 of 42 studies. Demonstrating a stable description. D3-outcomes were achieved through high-risk evaluations of all social media-based research articles due to a systematic lack of clinically validated reference standards clearly supporting the bias challenges identified earlier. D4-analysis was classified as unclear risk assessments in 36 out of 42 studies. Owing to the lack of external validation and fairness assessment, the surveyed literature shows that fairness in algorithms remains unexplored. The consistent lack of fairness assessment in the most widely surveyed literature suggests that computational fairness should be considered an essential evaluation standard, alongside predictive accuracy, in upcoming AI-based studies of depressive disorder detection. It is accompanied by a Supplementary Table S1, based on the table in Figure 5, which presents a summary of the percentage of studies included that rated the risk as low, high, or unclear in each domain.

## 5 Research gap

The survey brings out various pivotal research gaps that limit the present depressive disorder and postnatal depression detection frameworks. Existing approaches need a combination of modern deep learning frameworks, time-dependent analysis, and transfer learning methods to boost predictive performance. There remains a Scarcity of Cross-lingual, multi-source, cross-center data corpora, and few studies fuse multimodal data such as text, voice data, audio, facial features, biosignals, or neuroimaging features. The restriction in the machine learning model to capture sophisticated behavior patterns from the user, a detailed textual representation, is restricted by a natural language processing framework due to the shortage of data. Together, Feature extraction and fusion for multimodal data remain underdeveloped. Most importantly, model clarity and interpretability are rarely included, which slows health care uptake. Furthermore, most studies omit user behavior patterns, neglect to evaluate severity scores, and lack comparisons with benchmarking and advanced architecture. Even though postnatal depression detection has been the focus of many standalone investigations, comprehensive survey papers critically evaluate progress in data sets, attribute engineering, DL models and XAI techniques remain significantly scarce, where most of the studies reviewed include utilizing explainable AI as a Post-Hoc assessment and have not integrated explainability into their architecture development processes and its implementation for multimodal data types in postnatal data is remains predominantly un explored. There is a lack of longitudinal healthcare evaluation of whether XAI-based insights optimize healthcare decision-making or individual outcomes in the direction of clinical postpartum depression. Upcoming research focused on developing trustworthy maternal emotional well-being AI frameworks needs to prioritize fundamentally explainable models, incorporate heterogeneous XAI methods, and conduct clinical validation. These shortcomings highlight the importance of model improvement, scalability, and interpretability.

## 6 Conclusion

This review synthesized recent progress in postpartum depression detection and related depression detection to provide insights into techniques, limitations, and evaluation metrics used in mental disorders, so that it can be helpful for the improvement of early detection and diagnosis in maternal mental health using machine learning and deep learning. The most widely used deep learning techniques are CNN, GRU, LSTM, and BILSTM. In machine learning techniques, they have utilized most supervised ML algorithms.

The findings across all categories of predictive model performance are predominantly driven by data reliability and data modality, rather than model complexity alone. Additionally, lightweight models based on standardized clinical data tend to achieve predictive outcomes comparable to high-level complex models. Such results suggest that the field currently focuses on AI-based predictive performance over clinical relevance, a tendency that should be addressed in future studies.

These models achieve the elevated performance metrics in accuracy, sensitivity, and F1-Score. The area still encounters prominent limitations in research. The present study depends on limited or one-source data sets, lacks heterogeneity across languages and demographics, and frequently overlooks multimodal fusion data. These gaps are substantiated by the PROBAST+AI methodological quality risk of bias. Assessment was ranked as a considerable risk in the majority of studies using social media data due to a lack of clinically validated reference standards, and the model analysis domain is considered an unclear risk due to insufficient external validation and fairness assessment. Transparency remains underexplored, limiting healthcare implementation. Additionally, a limited number of studies integrate AI-derived interactional information or analyze prolonged emotional changes employing real-time or online frameworks.

Upcoming studies should focus on developing standardized, multilingual data collections, incorporating context-aware interactive datasets, strengthening model clarity via explainable AI techniques, and assessing models in clinical contexts. Real-time analysis will be facilitated by advances in low-complexity, efficient algorithms. Future investigations can incorporate chatbots and online platforms to predict severity and assess detection capabilities. Addressing these shortcomings will result in greater reliability, interpretability, and scalable platforms for initial PPD detection. These improvements will eventually support enhanced perinatal mental well-being outcomes.
