# Supplementary material for: Analysis of postpartum and other depression detection using machine learning and deep learning techniques: a systematic review
Source: Front Artif Intell. 2026 Aug 18;9:1849458. doi: 10.3389/frai.2026.1849458 (PMC13529931; doi:10.3389/frai.2026.1849458)
Supplement: Supplementary file 1 [file Table_1.DOCX]

**Supplementary materials**

Title: “Analysis of postpartum and other depression detection using Machine learning and deep learning techniques: A systematic review”

**Supplementary Table P1: Full search strategies**

| **Data source** | **Search strategy** |
| --- | --- |
| Science Direct (Elsevier) | (“Postpartum depression” OR “Postnatal Depression” OR “Perinatal Depression” OR ‘’Maternal Mental Health” OR “Pregnancy Related” OR “Depression” OR “Mental Health”) AND (“Machine Learning” OR “Deep Learning” OR “XAI” OR “Explainable AI”) |
| Springer Nature (Including BMC and Scientific Reports) | (“Postpartum depression” OR “Postnatal” OR “Perinatal Depression” OR ‘’Maternal Mental Health”) AND (“Machine Learning” OR “Deep Learning” OR “XAI”) |
| IEEE Xplore | (“Postpartum depression” OR “Postnatal” OR “Perinatal Depression”) AND (“Machine Learning” OR “Deep Learning” OR “XAI”) |
| Taylor & Francis | (“Postpartum depression”) AND (“Machine Learning” OR “Deep Learning” OR “XAI”) AND (“Depression”) |
| Wiley Online Library | (‘’Maternal Mental Health” OR “Postpartum depression” OR “Pregnancy Related” AND (“Machine Learning” OR “Deep Learning”) |
| WHO websites | Search browser topics related to “Depression” AND “Maternal Mental Health” (used for contextual and background statistics only) |
| PeerJ | “Depression” AND (“Machine Learning” OR “Deep Learning”) AND (“Social Media or Postpartum”) |
| Tech Science Press | “Depression detection” AND “Machine Learning OR Deep Learning” |

**Supplementary Table P2: List of Publications Included in this study**

This table provides a complete record of the publication venues for the 49 primary studies

included in this systematic review

| **Publications** | **Number of Included Studies** |
| --- | --- |
| Scientific Reports | 4 |
| Computer Methods and Programs in Biomedicine | 4 |
| Computers in Biology and Medicine | 3 |
| IEEE Access | 2 |
| IEEE Journal for Biomedical and Health Informatics | 2 |
| World Health Organization (web page) | 2 |
| BMC Pregnancy and Childbirth | 1 |
| IEEE Transactions on Computational Social Systems | 1 |
| PeerJ Computer Science | 1 |
| Journal of Medical Internet Research | 1 |
| Artificial Intelligence in Medicine | 1 |
| BMC Medical Informatics and Decision Making | 1 |
| Online Social Networks and Media | 1 |
| Personalized Medicine in Psychiatry | 1 |
| Applied Artificial Intelligence | 1 |
| Brain Informatics | 1 |
| International Journal of Cognitive Computing in Engineering | 1 |
| Translational Psychiatry | 1 |
| Network Modeling Analysis in Health Informatics and Bioinformatics | 1 |
| Intelligent Automation and Soft Computing | 1 |
| Engineering Applications of Artificial Intelligence | 1 |
| Procedia Computer Science | 1 |
| Complex & Intelligence Systems | 1 |
| Applied Soft Computing | 1 |
| BMJ | 1 |
| Information Fusion | 1 |
| Biomedical Signal Processing and Control | 1 |
| Computers in Biology and Chemistry | 1 |
| Journal of Translational Medicine | 1 |
| Computational Intelligence and Neuroscience | 1 |
| Smart Health | 1 |
| IEEE Transactions on Instrumentation and Measurement | 1 |
| System Science and Control Engineering | 1 |
| Measurement: Sensors | 1 |
| SLAS Technology | 1 |
| Journal of Affective Disorders | 1 |
| IEEE Transactions on Affective Computing | 1 |
| Neuro Image | 1 |
| Total | 49 |

**Supplementary Table P3: PRISMA 2020 Checklist**

| **Section and Topic** | **Item #** | **Checklist item** | **Location where item is reported** |
| --- | --- | --- | --- |
| **TITLE** | | |  |
| Title | 1 | Identify the report as a systematic review. | Page 1 |
| **ABSTRACT** | | |  |
| Abstract | 2 | See the PRISMA 2020 for Abstracts checklist. | Abstract (page 1) |
| **INTRODUCTION** | | |  |
| Rationale | 3 | Describe the rationale for the review in the context of existing knowledge. | Page 1-2 (Section 1) |
| Objectives | 4 | Provide an explicit statement of the objective(s) or question(s) the review addresses. | Page 2 ( Section 1) |
| **METHODS** | | |  |
| Eligibility criteria | 5 | Specify the inclusion and exclusion criteria for the review and how studies were grouped for the syntheses. | Page 4-5 (section 2.2, Table 1) |
| Information sources | 6 | Specify all databases, registers, websites, organisations, reference lists and other sources searched or consulted to identify studies. Specify the date when each source was last searched or consulted. | Page 4 (Section 2.1) |
| Search strategy | 7 | Present the full search strategies for all databases, registers and websites, including any filters and limits used. | Page 4 (Section 2.1)  Supplementary File 1 |
| Selection process | 8 | Specify the methods used to decide whether a study met the inclusion criteria of the review, including how many reviewers screened each record and each report retrieved, whether they worked independently, and if applicable, details of automation tools used in the process. | Page 5 (Section 2.3, Figure 3) |
| Data collection process | 9 | Specify the methods used to collect data from reports, including how many reviewers collected data from each report, whether they worked independently, any processes for obtaining or confirming data from study investigators, and if applicable, details of automation tools used in the process. | Page 4-6 (Section 2, 2.1, 2. 2, 2.3, Table 1) |
| Data items | 10a | List and define all outcomes for which data were sought. | Page 8-18 (Section 3.1 – 3.4) |
| Study risk of bias assessment | 11 | Specify the methods used to assess risk of bias in the included studies | Page 6 (Section 2.4) – PROBAST+AI |
| Effect measures | 12 | Specify for each outcome the effect measure(s) (e.g. risk ratio, mean difference) used in the synthesis or presentation of results. | N/A |
| Synthesis methods | 13 | Describe any methods used to tabulate or visually display results of individual studies and syntheses. | Page 7-20 (Section 3-4) |
|  | 13 a | Describe methods used to synthesize results, with rationale for the choice(s). | Page 7-20 (Section 3-4) |
| Reporting bias assessment | 14 | Describe any methods used to assess risk of bias due to missing results in a synthesis (arising from reporting biases). | Not reported |
| Certainty assessment | 15 | Describe any methods used to assess certainty (or confidence) in the body of evidence for an outcome. | N/A |
| **RESULTS** | | |  |
| Study selection | 16a | Describe the results of the search and selection process, from the number of records identified in the search to the number of studies included in the review, ideally using a flow diagram. | Page 5-6 (Section 2.3, Figure 3) |
|  | 16b | Cite studies that might appear to meet the inclusion criteria, but which were excluded, and explain why they were excluded. | Page 5-6 (Section 2.3, Figure 3) |
| Study characteristics | 17 | Cite each included study and present its characteristics. | Page 7-20 (Section 3.1 – 3.4); page 22 (Table 2-7) |
| Risk of bias in studies | 18 | Present assessments of risk of bias for each included study. | Page 6 (Section 2.4); page 25 (Figure 5); Supplementary Table S1 |
| Results of individual studies | 19 | For all outcomes, present rsults for each study | Page 7-20 (Section 3.1 – 3.4), (Table 2-6) |
| Results of syntheses | 20a | For each synthesis, briefly summarise the characteristics and risk of bias among contributing studies. | Page 7-20 (Section 3.1 – 3.4); (Section 4.2.1 – 4.2.3) |
| Reporting biases | 21 | Present assessments of risk of bias due to missing results (arising from reporting biases) for each synthesis assessed. | Not reported |
| Certainty of evidence | 22 | Present assessments of certainty (or confidence) in the body of evidence for each outcome assessed. | N/A |
| **DISCUSSION** | | |  |
| Discussion | 23a | Provide a general interpretation of the results in the context of other evidence. | Page 24-25 (Section 4.2.1 - 4.2.3); conclusion |
|  | 23b | Discuss any limitations of the evidence included in the review. | Page 7-20 (Section 3.1 – 3.4); page 24 (Section 4.2.3); conclusion |
|  | 23c | Discuss any limitations of the review processes used. | Page 26 (Conclusion) |
| **OTHER INFORMATION** | | |  |
| Registration and protocol | 24a | Provide registration information for the review, including register name and registration number, or state that the review was not registered. | Not reported |
|  | 24b | Indicate where the review protocol can be accessed, or state that a protocol was not prepared. | Not reported |
|  | 24c | Describe and explain any amendments to information provided at registration or in the protocol. | N/A |
| Support | 25 | Describe sources of financial or non-financial support for the review, and the role of the funders or sponsors in the review. | Page 27-Acknowledgements: Funding Statement |
| Competing interests | 26 | Declare any competing interests of review authors. | Page 27-Acknowledgements: Conflict of Interest Statement |
| Availability of data, code and other materials | 27 | Report which of the following are publicly available and where they can be found template data collection forms; data extracted from included studies; data used for all analyses; analytic code; any other materials used in the review. | Page 27- Data Availability Statement; Supplementary Material |
