# Supplementary material for: Analysis of postpartum and other depression detection using machine learning and deep learning techniques: a systematic review
Source: Front Artif Intell. 2026 Aug 18;9:1849458. doi: 10.3389/frai.2026.1849458 (PMC13529931; doi:10.3389/frai.2026.1849458)
Supplement: Supplementary file 2 [file Table_2.DOCX]

**Supplementary Table S1: PROBAST + AI (Risk of Bias assessment)**

This table summarises the detailed explanations of the Risk of Bias assessments for studies included in the present review.

- Assessment based on four domains
- Risk of bias D1, D2, D3, and D4
- Applicability: D1, D2, and D3
- Overall assessment for Risk of Bias and Applicability

| **Author** | **Risk of Bias** | | | | **Applicability** | | | **Overall** | |
| --- | --- | --- | --- | --- | --- | --- | --- | --- | --- |
|  | **D1: Participants & Data sources** | **D2: Predictors** | **D3: Outcome** | **D4: Analysis** | **D1: Participants** | **D2: Predictors** | **D3: Outcome** | **Risk of Bias** | **Applicability** |
| Group A: Postpartum Depression detection studies (EHR, questionnaire, Wearable) | | | | | | | | | |
| (Y. Zhang et al., 2021) | L | L | L | L | LC | LC | LC | L | LC |
| (Amit et al., 2021) | L | L | L | U | LC | LC | LC | U | LC |
| (Andersson et al., 2021) | L | L | L | U | UC | LC | LC | U | UC |
| (Oğur et al., 2023) | L | L | U | U | UC | LC | UC | U | UC |
| (Qi et al., 2025) | L | L | L | U | LC | LC | LC | U | LC |
| (Nasim et al., 2024) | H | L | U | U | HC | UC | HC | H | HC |
| (Shivaprasad et al., 2024) | H | L | U | H | H | U | H | H | HC |
| (Moreira et al., 2019) | U | L | L | U | UC | UC | UC | U | UC |
| (Kafi et al., 2026) | L | L | L | U | LC | LC | LC | U | LC |
| Group B: Postpartum depression detection (Multimodal, Deep Learning & NLP-based) | | | | | | | | | |
| (Gopalakrishnan et al., 2023) | U | L | U | U | HC | UC | UC | U | HC |
| (Lilhore et al., 2024) | H | L | U | H | HC | UC | HC | H | HC |
| (Gopalakrishnan et al., 2025) | L | L | L | L | LC | LC | LC | L | LC |
| (Krishnamurti et al., 2022) | L | L | L | U | LC | UC | LC | U | UC |
| (Wang, 2025) | L | L | U | U | UC | UC | UC | U | UC |
| (García-Méndez & de Arriba-Pérez, 2025) | U | L | U | U | HC | UC | HC | U | HC |
| Group C: General Depression Detection (Social media & Text-based) | | | | | | | | | |
| (Ahmad Wani et al., 2023) | L | L | H | U | HC | HC | HC | H | HC |
| (Figuerêdo et al., 2022) | L | L | H | U | HC | HC | HC | H | HC |
| (H. Zhang et al., 2023) | L | L | H | U | HC | HC | HC | H | HC |
| (Khafaga et al., 2023) | L | L | H | H | HC | HC | HC | H | HC |
| (Fkih et al., 2025) | H | L | H | U | HC | HC | HC | H | HC |
| (Kodati & Dasari, 2024) | L | L | H | U | HC | HC | HC | H | HC |
| (Akyol, 2023) | L | L | H | U | HC | UC | HC | H | HC |
| (J. Li et al., 2023) | H | L | H | U | HC | HC | HC | H | HC |
| (Karmen et al., 2015) | H | H | H | H | HC | HC | HC | H | HC |
| (Zogan et al., 2024) | L | L | H | U | HC | HC | HC | H | HC |
| (Vandana et al., 2023) | L | L | H | U | HC | HC | HC | H | HC |
| Group D: General Depression Detection (Multimodal, EEG, MRI, Wearable) | | | | | | | | | |
| (Patil et al., 2024) | L | L | U | L | UC | UC | UC | U | UC |
| (Patil et al., 2023) | U | L | U | U | UC | UC | UC | U | UC |
| (Zhou et al., 2024) | L | L | L | U | UC | U | LC | U | UC |
| (Seal et al., 2021) | U | L | U | U | UC | UC | UC | U | UC |
| (Sun et al., 2023) | L | L | L | U | UC | UC | LC | U | UC |
| (Sarwar et al., 2024) | U | L | U | L | UC | UC | UC | U | UC |
| (Kaur et al., 2022) | U | L | U | U | UC | UC | UC | U | UC |
| (Saif Alghawli & Taloba, 2022) | H | L | U | U | HC | UC | UC | H | HC |
| (Zhao & Tlachac, 2025) | U | L | U | L | UC | UC | UC | U | UC |
| (Benito et al., 2024) | L | L | L | U | UC | LC | UC | U | UC |
| (Zhu et al., 2023) | L | L | L | L | UC | LC | LC | L | UC |
| (Dang et al., 2024) | L | L | L | L | LC | LC | LC | L | LC |
| (Hwang et al., 2025) | L | L | L | U | UC | UC | UC | U | UC |
| (Rafiei et al., 2022) | L | L | L | U | UC | UC | UC | U | UC |
| (Z. Li et al., 2022) | U | L | U | U | U | UC | UC | U | UC |
| (Dunjic-Kostic et al., 2025) | L | L | U | U | UC | UC | UC | U | UC |
| Risk of Bias: L = Low Risk (criterion met), U = Unclear Risk (Partially met or insufficient information), H = High Risk (significant methodological flow)  Applicability: LC = Low Concern (matches review target), UC = Unclear Concern, HC = High Concern (not match clinical target) | | | | | | | | | |
